# An Extract from the Plant *Deschampsia antarctica* Protects Fibroblasts from Senescence Induced by Hydrogen Peroxide

**DOI:** 10.1155/2017/2694945

**Published:** 2017-08-15

**Authors:** Ana Ortiz-Espín, Esther Morel, Ángeles Juarranz, Antonio Guerrero, Salvador González, Ana Jiménez, Francisca Sevilla

**Affiliations:** ^1^Department of Stress Biology and Plant Pathology, Centro de Edafología y Biología Aplicada del Segura, Consejo Superior de Investigaciones Científicas, Campus de Espinardo, 30100 Murcia, Spain; ^2^Department of Biology, Faculty of Sciences, Universidad Autónoma de Madrid, 28049 Madrid, Spain; ^3^IFC SA-Industrial Farmaceutica Cantabria, 28043 Madrid, Spain; ^4^Department of Medicine and Medical Specialties, Universidad de Alcalá de Henares, 28805 Madrid, Spain; ^5^Dermatology Service, Memorial Sloan-Kettering Cancer Center, New York, NY, USA

## Abstract

The Antarctic plant *Deschampsia antarctica* (DA) is able to survive in extreme conditions thanks to its special mechanism of protection against environmental aggressions. In this work, we investigated whether an aqueous extract of the plant (EDA) retains some of its defensive properties and is able to protect our skin against common external oxidants. We evaluated EDA over young human fibroblasts and exposed to H_2_O_2_, and we measured cell proliferation, viability, and senescence-associated *β*-galactosidase (SA-*β*-Gal). We also tested the expression of several senescence-associated proteins including sirtuin1, lamin A/C, the replicative protein PCNA, and the redox protein thioredoxin 2. We found that EDA promoted *per se* cell proliferation and viability and increased the expression of anti-senescence-related markers. Then, we selected a dose of H_2_O_2_ as an inductor of senescence in human fibroblasts, and we found that an EDA treatment 24 h prior H_2_O_2_ exposure increased fibroblast proliferation. EDA significantly inhibited the increase in SA-*β*-Gal levels induced by H_2_O_2_ and promoted the expression of sirtuin 1 and lamin A/C proteins. Altogether, these results suggest that EDA protects human fibroblasts from cellular senescence induced by H_2_O_2_, pointing to this compound as a potential therapeutic agent to treat or prevent skin senescence.

## 1. Introduction

Aging is a complex biological process that involves intrinsic genetic variations and external factors such as nutrition, diseases, or environmental conditions [1]. Hydrogen peroxide (H_2_O_2_) is an oxidant agent that induces a typical senescence called “stress-induced premature senescence” (SIPS) when applied exogenously *in vitro.* SIPS shares some features with natural or replicative senescence [2–4], including changes in the morphology of the cells, decreased cell proliferation, and DNA synthesis and increased SA-*β*-Gal levels [5, 6]. A previous work has shown that cellular senescence in human diploid fibroblasts is accompanied by a decrease in the expression of proliferating cell nuclear antigen (PCNA), a protein involved in replication and cell cycle [7, 8]. This effect on PCNA correlates with a decrease in cell proliferation as well as lower levels of several proteins involved in metabolism. Some of these are sirtuins (Sirt), which are nicotinamide adenine dinucleotide- (NAD^+^-) dependent histone deacetylases. The Sirt family includes several members localized in different subcellular compartments: the nuclei (Sirt1, Sirt2, Sirt6, and Sirt7), cytoplasms (Sirt1 and Sirt2), and mitochondria (Sirt3, Sirt4, and Sirt5) [9, 10]. Stress downregulates Sirt1, promoting acetylation of p53 and the acquisition of a premature senescence-like phenotype [11–16]. Conversely, overexpression of Sirt1 prevents cells from physiological alteration toward senescence [13].

Senescence also promotes changes in the architecture of the nucleus of mammalian cells. Lamins are nucleoskeletal proteins that define the structure of the nucleus. Some reports reveal that contents of lamin A/C (LmnA/C) show changes in expression along organism life [17, 18]. A reduction of LmnA/C has been observed in osteoblasts of old mice as compared to those of young mice [17].

During senescence, an increase of free radicals induces oxidative damage in almost every cell compartment [19]. Redox proteins such as thioredoxins (Trxs) reduce free radical-promoted damage in stress-induced premature senescence cells [20–22] through activation of the p16 and p53 tumor suppressor pathways [17, 22] or reducing indirectly the ROS levels [23]. Mammalian cells express two Trx isoforms, the cytosolic-nuclear Trx1 and the mitochondrial Trx2. Mitochondria is the main source of ROS in senescence, and Trx2 is one of the most important protein-regulating oxidative stress in this organelle [24].

Research of natural substances that can delay skin aging has been the object of increasing interest in the last few years [4, 25, 26]. In this regard, *Deschampsia antarctica* (DA) is an Antarctic plant able to live under high-solar irradiation, high-salinity and high-oxygen concentrations, low temperature, and extreme dryness. Some of the DA defensive properties have been reported *in vivo* in human cells, and an extract from the plant (EDA) prevents UV-induced human photoaging (patent US 8.357.407 B2). In order to get more information about the potential benefits of EDA over skin aging induced by external oxidants agents, we have evaluated the effect of different treatments of EDA on the senescence process of young fibroblasts from human foreskin induced by several H_2_O_2_ concentrations. Cell proliferation and morphological measurements together with quantification of the levels of PCNA as marker of DNA replication; the senescence markers *β*-galactosidase, Lamin A/C, and Sirt1; and the redox protein Trx2 revealed a protective effect of EDA, positioning it as a therapeutic candidate to counteract oxidation-dependent cell senescence induced by H_2_O_2_ exposure.

## 2. Materials and Methods

### 2.1. Reagents

*Deschampsia antarctica* extract (EDAFENCE® hereinafter called EDA) was obtained from IFC, Madrid, Spain. Briefly, dry green leaves, harvested from cultured *Deschampsia antarctica* plants in defined conditions, were milled and extracted by percolation with water at 40–60°C, during 4–6 hours. The extract was filtered through a 1 *μ*m filter and lyophilized.

### 2.2. Cell Culture and Treatments

Senescent human foreskin fibroblasts (SHFF) were derived from primary human foreskin fibroblasts (HFF) by repeated passages. Stress-induced premature senescence fibroblasts (SIPSF) were obtained by exposure to H_2_O_2_. Primary young fibroblasts were considered of less than 20 population doublings (PD), presenescent between 21 and 29 PD, and senescent cultures at 30 PD or later. The cells were propagated in F-25 flasks. All experiments were done with young (13 PD), presenescent (26 PD), and senescent (45 PD) fibroblasts. The medium used was Dulbecco's modified Eagle's medium (DMEM) supplemented with 4.5 g/L glucose, 10% (*v*/*v*) fetal calf serum (FCS), 50 *μ*g/mL penicillin, and 0.2 M L-glutamine. Cells were incubated at 37°C in an atmosphere containing 5% CO_2_. When cells reached the confluency, they were subcultured using a solution containing 0.25% trypsin and 0.02% EDTA.

For the EDA treatment, this compound was dissolved in sterile water under agitation during 30 min at 25°C and sterilized by filtration through 0.22 *μ*m syringe filters to a final concentration of 10 mg/mL. EDA treatments were done at 0.3 mg/mL, 0.5 mg/mL, and 1 mg/mL. HFF cultures were treated at different times: 24 h after seeding during 24 h (condition 1) and 48 h (condition 3) and 48 h after seeding during 24 h (condition 2), (Supplemental Figure S1 available online at https://doi.org/10.1155/2017/2694945). For H_2_O_2_-induced premature senescence, cells at 48 h after seeding were treated with different H_2_O_2_ concentrations (100 *μ*M, 150 *μ*M, and 200 *μ*M) for different times (30 min, 1 h, and 2 h). In these assays, cells were incubated with 0.3, 0.5, and 1 mg/mL EDA during 24 h before H_2_O_2_ treatment (PRE-treatment), after H_2_O_2_ treatment (POST-treatment), and before and after the treatment (PRE-POST-treatment), (see Supplemental Figure S2). In all cases, the treatment with H_2_O_2_ was done only with basalt medium and H_2_O_2_. EDA was removed from the medium immediately before the H_2_O_2_ exposition.

### 2.3. Cell Viability and Proliferation Assays

Cell viability was measured by the MTT reduction assay and cell proliferation was assayed using the crystal violet staining. Cells were seeded in a 96-well plate at a density of 3.5 × 10^3^cells/well. At the measure point, MTT was added at 0.5 mg/mL for 2 h at 37°C. The result formazan product was solubilized in DMSO under agitation during 30 min, and absorbance was measured at 570 nm and 690 nm (background). Crystal violet was prepared at 0.1% *w*/*v* in 2% ethanol. Previously, cells were fixed with Carnoy staining (methanol: acetic acid in 3 : 1 (*v*/*v* proportion)) during 5 min, and after being completely dried, they were incubated with the crystal violet solution during 30 min at 37°C. Cells were washed several times with water (until rinses were clear). Crystal violet was extracted from cells with 40% (*v*/*v*) methanol under agitation during 30 min and absorbance was measured at 570 nm.

### 2.4. SA Beta Galactosidase Level

The *β*-Gal level was determined as described elsewhere [27]. Briefly, subconfluent cultures were previously fixed for 5 min with 0.2% (*v*/*v*) glutaraldehyde and 2% (*v*/*v*) formaldehyde and afterwards rinsed with PBS. For the fluorescent assay, cells were incubated with a reaction solution containing 0.2 M citrate Na_2_HPO_4_ pH 5.0, 6.4 mM MgCl_2_, 100 mM potassium hexacyanoferrate (II) trihydrate, 100 mM potassium hexacyanoferrate (III), 5 M NaCl, and 2 mM fluorescein di-*β*-galactopyranoside (FDG) solubilized in DMSO. Fixed fibroblasts were incubated with the reaction solution during 16–20 h at 37°C. Fluorescence was measured at 485/525 nm excitation/emission wavelengths. For the colorimetric assay, FDG was substituted by 100 *μ*M X-Gal (5-bromo-4-chloro-3-indolyl *β*-D-galactopyranoside). Positive cells were detected as blue stained, under a standard light microscopy. A total of 1000 cells were counted in five random fields on culture plates to determine the percentage of *β*-Gal-positive cells [27].

### 2.5. Western Blot Analysis

Subconfluent cultures were washed with PBS 1× and then lysed with RIPA buffer containing phosphatase cocktail 2 and protease inhibitor cocktail (Sigma, St Louis, MI, U.S.A.). The sample protein contents were adjusted to the same protein concentration measured by the BCA protein assay reagent (Pierce, Rockford, IL, U.S.A.), mixed with Laemmli sample buffer containing 50 mM DTT and boiled for 5 min. Afterwards, 80 *μ*g of each sample was subjected to electrophoretic separation in 6–12% SDS-PAGE. Gels were then transferred to a nitrocellulose membrane (0.2 *μ*m pore, Millipore, Bedford, MA, U.S.A.) for 10 minutes using the Trans-Blot Turbo Transfer System (Bio-Rad, California, USA). Membranes were stained with Ponceau S staining solution (0.1% (*w*/*v*) Ponceau S in 5% (*v*/*v*) acetic acid) to control the loading, and after bleaching, membranes were blocked with 1% BSA in Tris-buffered saline (TBS: 25 mM Tris-HCl pH 7.5, 150 mM NaCl). The membranes were incubated in TBS containing 0.1% Tween-20 and 1% BSA (TBST) and the following specific antibodies: goat polyclonal Actin-C (1 : 1000), mouse monoclonal PCNA (1 : 3000), goat polyclonal Trx2 (1 : 1000), goat polyclonal Sirt1 (1 : 1000), and goat polyclonal LmnA/C (1 : 1000). As secondary antibodies, we used HRP monoclonal antibody anti-mouse Ig G (1 : 6000) and HRP polyclonal anti-goat IgG (1:1000). Membranes were incubated with primary antibodies overnight at 4°C, and after being washed with TBST, secondary antibody was used to incubate the membranes 1 h at room temperature. Detection of bands was performed using ECL Plus Western blotting detection system (GE Healthcare, Hertfordshire, U.K.). To quantify the bands we applied Quantity One software-based analysis (Bio-Rad). All antibodies used were from Santa Cruz Biotechnology.

### 2.6. Microscopic Observations and Statistical Analysis

Microscopic observations and photographs were performed in a Nikon Mod. Diaphot-TMD photomicroscope equipped with a HBO 100 W mercury lamp and the corresponding filter sets for fluorescence microscopy: green (545 nm, exciting filter BP 545). Data were expressed as mean ± S.E. The statistical significance was determined using the analysis of variance (ANOVA) showed by Duncan post hoc test (*p* < 0.05) using SPSS software (IBM^R^ SPSS^R^ Statistics 19).

## 3. Results

### 3.1. EDA Affects Cell Proliferation and Viability of HFF Cells

**Figure 1:**
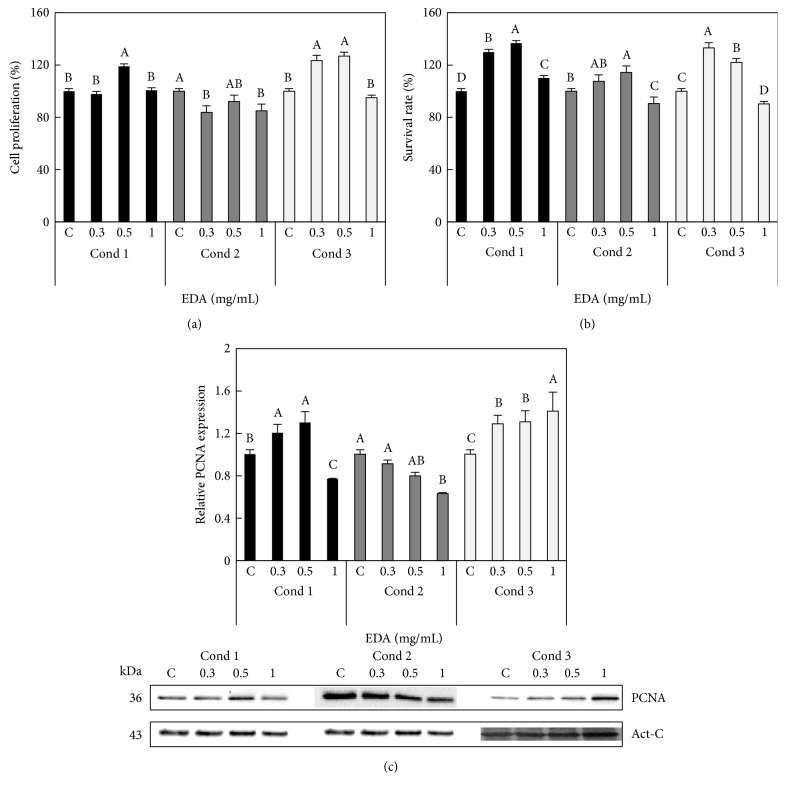
Effect of EDA in cell proliferation and viability of HFF cells. (a) Cell proliferation and (b) survival rate of control (C, untreated EDA cells) and EDA-treated cells with 0.3, 0.5, and 1 mg/mL. (c) Densitogram (upper panel) and Western blot (lower panel) of PCNA in control and EDA-treated cells. Act-C is used as loading control. The time of EDA treatment was 24 h after seeding during 24 h (Cond 1), 48 h after seeding during 24 h (Cond 2), and 24 h after seeding during 48 h (Cond 3). See more details in Supplemental Figure S1. Each bar represents the mean ± S.E. of three replicates from three independent experiments, and samples that do not have a common letter are significantly different in each condition by Duncan's test at *P* < 0.05.

To test whether EDA affected the proliferation of HFF cells (13 PD), we incubated cultures after 24 h and/or 48 h from seeding with different concentrations of EDA (0, 0.3, 0.5, and 1 mg/mL), as it is shown in Supplemental Figure S1. In all cases, we evaluated the different parameters after 72 h postseeding. In condition 1, only 0.5 mg/mL EDA increased cell proliferation whereas in condition 3, 0.3 mg/mL and 0.5 mg/mL increased this parameter. Both EDA concentrations also increased the survival rate in conditions 1 and 3, and 0.5 mg/mL increased it also in condition 2. Higher concentrations of EDA (1 mg/mL) decreased survival in conditions 2 and 3 but increased in condition 1 (Figures 1(a) and 1(b)).

We also evaluated cell proliferation indirectly by measuring the levels of PCNA, an important marker of cell replication. We found that in conditions 1 and 3, 0.3 and 0.5 mg/mL EDA induced a significant increase in the PCNA level compared to that in the control conditions. Nevertheless, higher EDA concentrations (1 mg/mL) decreased PCNA levels in conditions 1 and 2 but increased it in condition 3 (Figure 1(c)).

These results indicate that cell proliferation, survival rate, and PCNA levels are in agreement with the fact that cell proliferation of HFF cells increased with an EDA treatment of 0.3 and 0.5 mg/mL in both conditions 1 and 3.

### 3.2. EDA Treatment Promotes Expression of Both Sirt1 and LmnC and Decreases SA-*β*-Gal

We assessed the effect of EDA on the levels of several senescence markers in HFF cells (13 PD). In this regard, the process of senescence is characterized by an increase of SA-*β*-Gal-positive cells and by changes in the expression of known senescence markers such as Sirt1 and LmnA/C.

**Figure 2:**
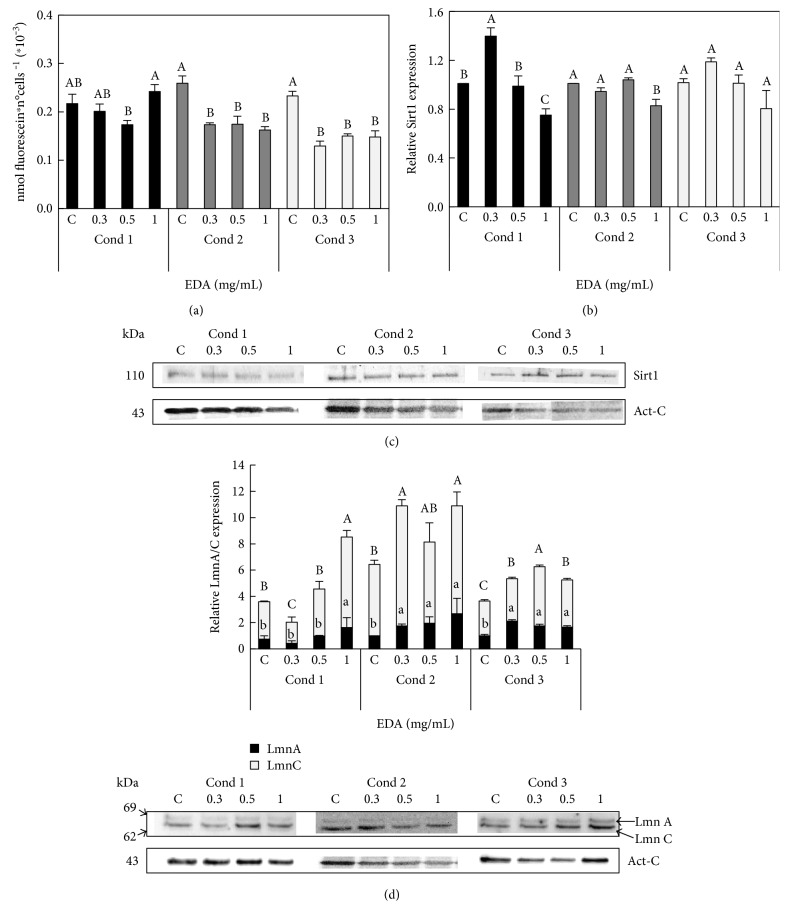
Effect of EDA in SA-*β*-Gal levels and protein contents of Sirt1 and Lmn A/C of HFF cells. (a) SA-*β*-Gal levels were measured as nmol of fluorescein by number of cells in control (C, untreated EDA cells) and EDA-treated cells with 0.3, 0.5, and 1 mg/mL. (b) Densitogram of Sirt1 in control and EDA-treated cells. (c) Western blot of Sirt1 in control and EDA-treated cells. (d) Densitogram (upper panel) and Western blot for LmnA/C (lower panel) in control and EDA-treated cells. Capital letters indicate statistical comparative analysis among LmnA data and lowercase letters among LmnC. Act-C is used as loading control. The time of EDA treatment was 24 h after seeding during 24 h (Cond 1), 48 h after seeding during 24 h (Cond 2), and 24 h after seeding during 48 h (Cond 3). See more details in Supplemental Figure S1. Each bar represents the mean ± S.E. of three replicates from three independent experiments, and samples that do not have a common letter are significantly different in each condition by Duncan's test at *P* < 0.05.

The addition of EDA to HFF caused a decrease in the SA-*β*-Gal level. In condition 1, any of the EDA concentrations decreased the SA-*β*-Gal level; however, all EDA concentrations were able to decrease SA-*β*-Gal in conditions 2 and 3 (Figure 2(a)). We next tested whether EDA treatments were able to induce expression of Sirt1, a marker of metabolism status and senescence. Western blot showed only an increase in Sirt1 protein content at 0.3 mg/mL EDA in condition 1 (Figures 2(b) and 2(c)). On the other hand, the nucleoskeleton protein LmnA/C increased at EDA doses of 1 mg/mL in condition 1 but increased at all EDA concentrations tested in conditions 2 and 3 (Figure 2(d)).

These results showed that different EDA treatments induced a decrease in markers of basal senescence including higher levels of LmnA/C and Sirt1 and a decrease in the SA-*β*-Gal level (Figure 2).

### 3.3. H_2_O_2_ Decreases Cell Proliferation and Survival

**Figure 3:**
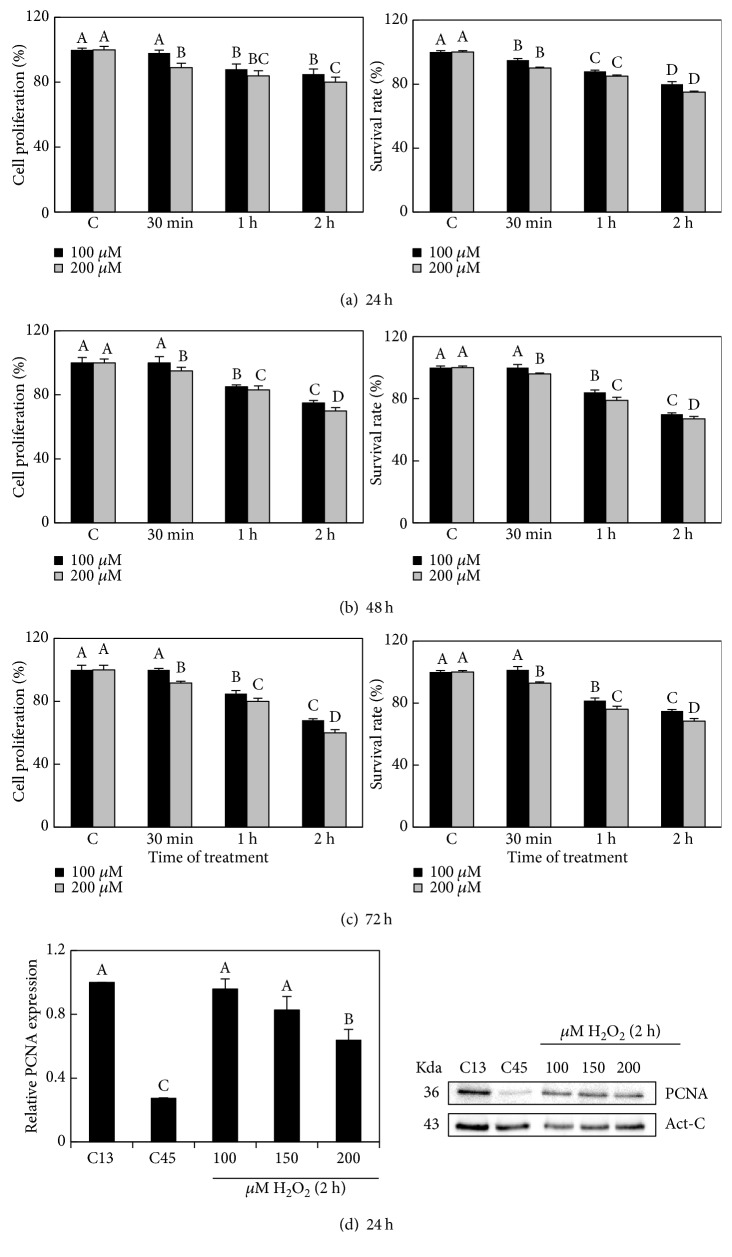
Effect of H_2_O_2_ in cell proliferation and survival rate of HFF cells. Cell proliferation and survival rate of control (H_2_O_2_-unexposed cells) and cells exposed to 100 and 200 *μ*M H_2_O_2_ during 30 min, 1 h, and 2 h. Measures were done at 24 h (a), 48 h (b), and 72 h (c) after H_2_O_2_ recovery. (d) Densitogram (left panel) and Western blot (right panel) of PCNA in controls (C13, cells of 13 PD (HFF) and C45, cells of 45 PD (SHFF)), and cells exposed to 100, 150, and 200 *μ*M H_2_O_2_ during 2 h and after 24 h of recovery. Capital letters indicate statistical comparative analysis among data from 100 *μ*M H_2_O_2_ treatment and lowercase letters among the 200 *μ*M H_2_O_2_ treatment. Each bar represents the mean ± S.E. of three replicates from three independent experiments, and samples that do not have a common letter are significantly different by Duncan's test at *P* < 0.05.

In order to establish a condition of extrinsic senescence, we evaluated the effect of different H_2_O_2_ concentrations over cell proliferation, viability, and associated senescence markers described in the previous section. Cell cultures (13 PD) were treated 48 h after seeding (50% of confluency) with different doses of H_2_O_2_ (100 and 200 *μ*M) for different times (30 min, 1 h, and 2 h). We performed the analysis at 24 h, 48 h, and 72 h after H_2_O_2_ exposure. A decrease in cell proliferation was observed at both H_2_O_2_ concentrations applied for 1 h and 2 h treatments. This effect was noticeable 24 h after H_2_O_2_ recovery, becoming more pronounced at 72 h. Data obtained from the survival rate were in agreement with these results (Figures 3(a), 3(b), and 3(c)). As expected, PCNA levels decreased significantly compared to those of control (C13), but only in cells treated with 200 *μ*M H_2_O_2_ for 2 h and after 24 h of H_2_O_2_ recovery (Figure 3(d)).

In conclusion, these results indicate that a range of 100–200 *μ*M H_2_O_2_ applied during 1-2 h decreased cell proliferation and viability in human fibroblasts in a sustained manner from 24 h to 72 h of recovery from the H_2_O_2_ treatment.

### 3.4. H_2_O_2_ Increases the Expression of Senescence-Related Proteins

**Figure 4:**
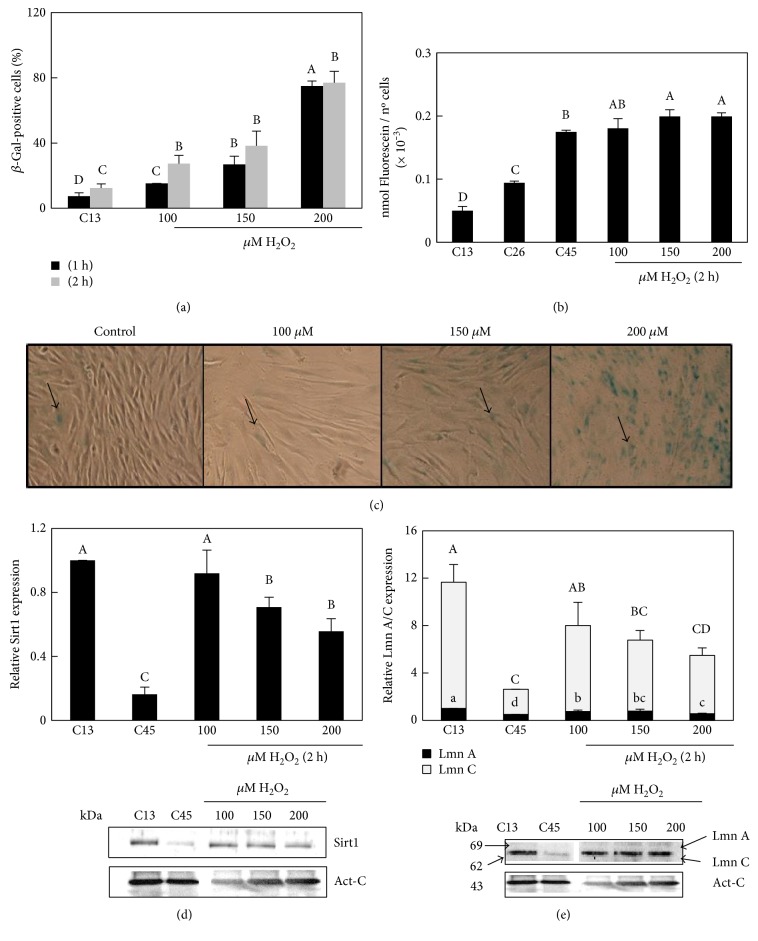
Effect of H_2_O_2_ treatment in the SA-*β*-Gal level and protein content of Sirt1 and LmnA/C of HFF cells. (a) The SA-*β*-Gal level measured as a number of blue cells in control (C13, H_2_O_2_-unexposed cells) and cells exposed to 100, 150, and 200 *μ*M H_2_O_2_ during 1 and 2 h and after 24 h of H_2_O_2_ recovery. Capital letters indicate statistical comparative analysis among data from 1 h H_2_O_2_ treatment and lowercase letters among 2 h H_2_O_2_ treatment (b) The *β*-Gal level measured as nmol of fluorescein per number of cells in controls (H_2_O_2_-unexposed cells, C13: 13 PD; C26: 26 PD; and C45: 45 PD) and cells exposed to 100, 150, and 200 *μ*M H_2_O_2_ during 2 h and after 24 h of recovery. (c) Cells stained with the chromogenic substrate 5-bromo-4-chloro-3-indolyl-beta-d-galactopyranoside (X-gal) prior to 100, 150, and 200 *μ*M H_2_O_2_ exposition and after 24 h of H_2_O_2_ recovery. (d, e) Densitogram (upper panel) and Western blot for Sirt1 and LmnA/C (lower panel) in controls (H_2_O_2_-unexposed cells: C13, C45) and H_2_O_2_-exposed cells during 2 h and after 24 h H_2_O_2_ recovery. Panel LmnA/C; capital letters indicate statistical comparative analysis among LmnA data and lowercase letters among LmnC. Act-C is used as loading control. Each bar represents the mean ± S.E. of three replicates from three independent experiments, and samples that do not have a common letter are significantly different by Duncan's test at *P* < 0.05.

We next analyzed the effect of H_2_O_2_ on the onset of the extrinsic senescence in HFF. Different H_2_O_2_ doses were used and we measured the SA-*β*-Gal level by colorimetric and fluorescence assays. Hydrogen peroxide induced an increase in the percentage of positive SA-*β*-Gal cells (blue cells) at every dose assayed, particularly at the highest one (200 *μ*M H_2_O_2_) (Figure 4(a)), but we were able to detect this increase only 48 h after H_2_O_2_ recovery. As an alternative measurement, we quantified SA-*β*-Gal using the fluorescence reagent FDG. After 24 h of H_2_O_2_ recovery, (see Material and Methods) all hydrogen peroxide concentrations assayed showed an increase in SA-*β*-Gal levels (Figure 4(b)). The sensitivity of this method affords us to detect SA-*β*-Gal in hydrogen peroxide cultures, 24 h before, than with the colorimetric method. However, the differences in SA-*β*-Gal levels between H_2_O_2_ treatments were more evident 48 h after recovery as it is shown in the colorimetric assay.

We next tested whether H_2_O_2_ exposure modulated expression of Sirt1 in HFF cells. We found that 150 and 200 *μ*M H_2_O_2_ decreased Sirt1 protein content compared to control cells from 13 PD (C13, Figure 4(d)). Regarding lamins, we found that cell cultures exposed to 150 and 200 *μ*M of hydrogen peroxide decreased around two fold levels of LmnA/C compared to those of the HFF control (Figure 4(e)).

In summary, our results are consistent with H_2_O_2_ inducing a senescent phenotype and they allowed us to determine an optimal dose and timing (200 *μ*M for 2 h).

### 3.5. A Treatment of H_2_O_2_ Induces Trx2 Expression

**Figure 5:**
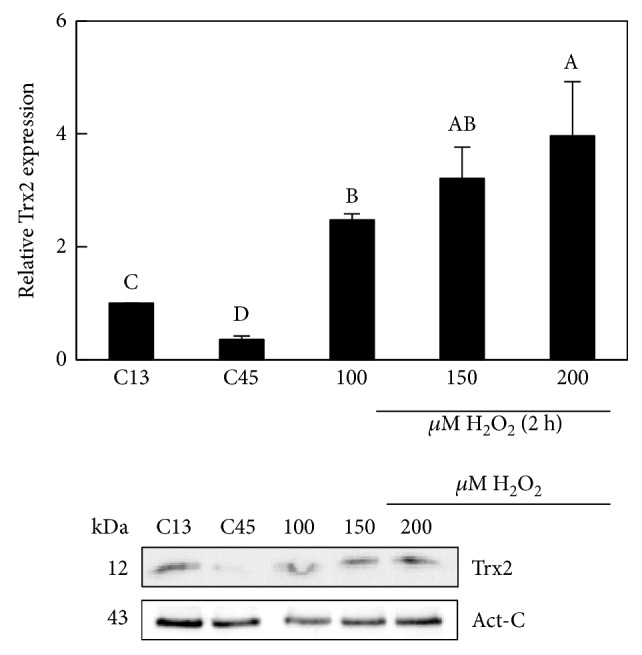
Effect of H_2_O_2_ in protein contents of Trx2 of HFF cells. Densitogram (upper panel) and Western blot (lower panel) of Trx2 in control (unexposed H_2_O_2_ cells: C13, C45) and H_2_O_2_-exposed cells during 2 h and after 24 h H_2_O_2_ recovery. Act-C is used as loading control. Each bar represents the mean ± S.E. of three replicates from three independent experiments, and samples that do not have a common letter are significantly different by Duncan's test at *P* < 0.05.

Trx2 is an important mitochondrial redox protein involved in different processes including apoptosis, cell cycle, and ROS response. Parallel to the previous assays, we also tested the Trx2 protein content in HFF (13 PD, C13), SHFF (45 PD, C45), and H_2_O_2_-exposed HFF cultures (SIPSF), (Figure 5). The results showed a decrease of Trx2 in SHFF although its level increased significantly (*P* < 0.05) after the exposition of HFF to 100, 150, and 200 *μ*M H_2_O_2_ for 2 h compared to unexposed HFF cells.

### 3.6. EDA Prevents Loss of Cell Viability in H_2_O_2_-Exposed Cells

**Figure 6:**
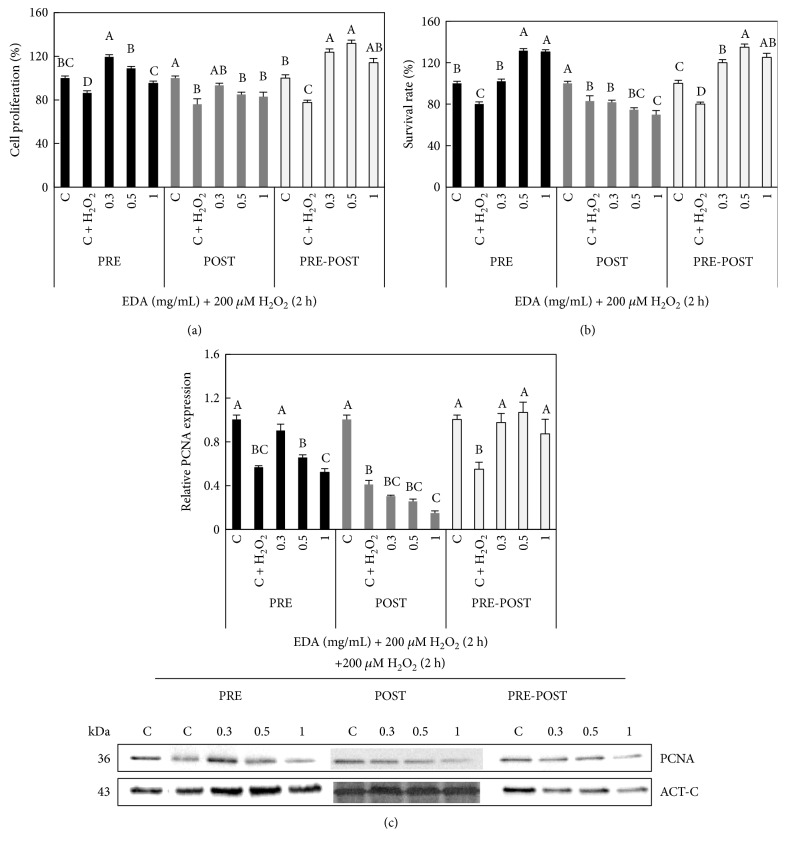
Effect of EDA in cell proliferation and viability of SIPSF cells. (a) Cell proliferation and (b) survival rate of control (C, unexposed HFF cells, neither EDA nor H_2_O_2_), control 200 *μ*M H_2_O_2_-exposed cells (C + H_2_O_2_), and H_2_O_2_-exposed cells treated with 0.3, 0.5, and 1 mg/mL EDA. (c) Densitogram (upper panel) and Western blot (lower panel) of PCNA in control (C, unexposed HFF cells, neither EDA nor H_2_O_2_), control 200 *μ*M H_2_O_2_-exposed cells (C + H_2_O_2_), and H_2_O_2_-exposed cells treated with 0.3, 0.5, and 1 mg/mL EDA. The time of EDA treatment was 24 h before H_2_O_2_ exposition (PRE), just after H_2_O_2_ exposition (POST), and after and before H_2_O_2_ exposition (PRE-POST). Each bar represents the mean ± S.E. of three replicates from three independent experiments, and samples that do not have a common letter are significantly different in each condition (PRE, POST, and PRE-POST) by Duncan's test at *P* < 0.05.

To assess the ability of EDA to protect against oxidative senescence-related damage or to repair its deleterious effects, we assessed different concentrations of EDA (0, 0.3, 0.5, and 1 mg/mL) and different incubation times in HFF, measuring proliferation and survival rate. HFF cells were treated with different concentrations of EDA prior to H_2_O_2_ exposure (PRE-condition) and/or after H_2_O_2_ exposition (PRE-POST condition and POST-condition, resp.). The evaluation of the different parameters was carried out 24 h after recovery from the H_2_O_2_ treatment (see supplemental Figure S2). As it can be seen in Figures 6(a) and 6(b), an EDA treatment (0.3, 0.5, and 1 mg/mL) prior H_2_O_2_ exposure and prior and after H_2_O_2_ exposure (PRE-POST-treatment) increased both cell proliferation and survival rate, even though there are over values found in control conditions (C) (approximately 10–20% increase).

We also found that the levels of PCNA correlated well with the data on survival and proliferation. We noted that PRE-treatment of 0.3 mg/mL EDA increased PCNA levels of SIPSF cells compared with those of control H_2_O_2_-exposed cells. Besides, the PRE-POST treatment increased PCNA levels in all EDA doses (0.3, 0.5, and 1 mg/mL). By contrast, the addition of EDA (0.3, 0.5 mg/mL) only after H_2_O_2_ treatment neither induced changes in cell proliferation nor improved their survival rate (Figure 6(b)). Moreover, the higher EDA doses (1 mg/mL) decreased cell proliferation, survival rate, and PCNA protein content (Figure 6).

### 3.7. EDA Modulates the Expression of Senescence Proteins in H_2_O_2_-Exposed Cells

**Figure 7:**
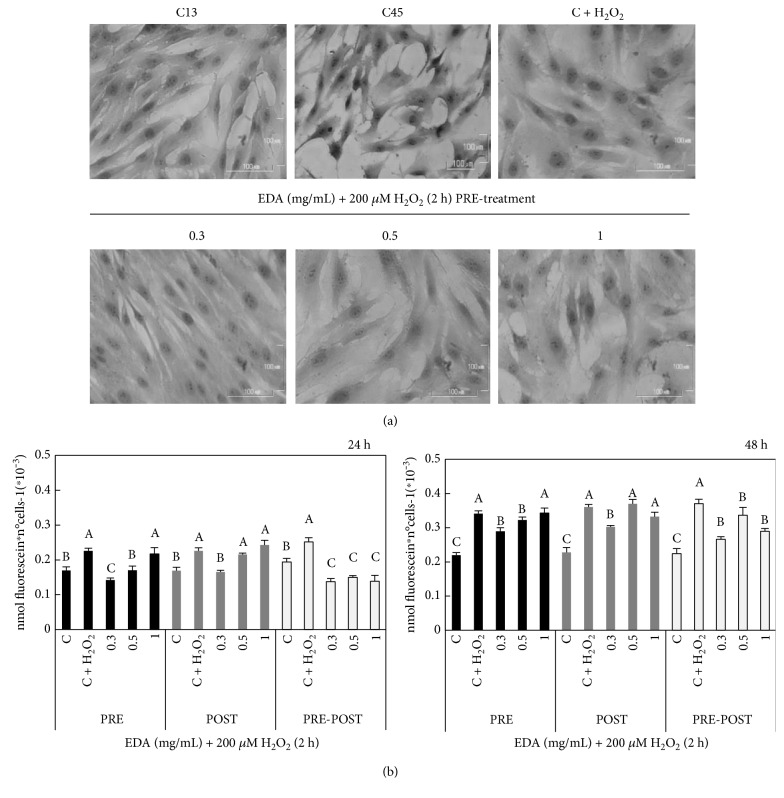
Effect of EDA in cellular morphology and SA-*β*-Gal of SIPSF cells. (a) SA-*β*-Gal was measured as nmol of fluorescein per number of cells in control (C, unexposed HFF cells), control 200 *μ*M H_2_O_2_-exposed cells (C + H_2_O_2_), and H_2_O_2_-exposed cells treated with 0.3, 0.5, and 1 mg/mL EDA measured at 24 h and 48 h after H_2_O_2_ exposition. (b) Cellular morphology in control cells (C13, cells from 13 PD; C45, cells from 45 PD; and C + H_2_O_2_) and H_2_O_2_-exposed cells treated with 0.3, 0.5, and 1 mg/mL EDA previous H_2_O_2_ exposure and 24 h after H_2_O_2_ recovery (lower line). The time of EDA treatment was 24 h before H_2_O_2_ exposition (PRE), just after H_2_O_2_ exposition (POST), and after and before H_2_O_2_ exposition (PRE-POST). Each bar represents the mean ± S.E. of three replicates from three independent experiments, and samples that do not have a common letter are significantly different in each condition (PRE, POST, and PRE-POST) by Duncan's test at *P* < 0.05.

Senescence causes profound changes in cell morphology. Hence, we tested whether the morphological changes promoted by H_2_O_2_ could be prevented or reverted by the EDA treatments. Morphological observations after crystal violet staining indicated that control SIPSF cells (C + H_2_O_2_) presented a more evident polyhedral morphology and were larger compared to that of control HFF cells (C13), which showed the characteristic spindle shape (Figure 7(a)). The morphology of cells treated with 0.3 mg/mL EDA prior H_2_O_2_ exposure was similar to H_2_O_2_-unexposed cells (control C13). However, treatments at higher doses (0.5 and 1 mg/mL EDA) did not cause substantial changes compared to those of control SIPSF cells (Figure 7(a)).

Also, PRE-treatment with 0.3 and 0.5 mg/mL EDA prior to H_2_O_2_ exposition significantly decreased H_2_O_2_-induced SA-*β*-Gal (*P* < 0.05) at 24 h and 48 h after H_2_O_2_ recovery (Figure 7(b)). Furthermore, EDA POST-treatment decreased SA-*β*-Gal (*P* < 0.05), but only at 0.3 mg/mL EDA doses. Noteworthy, PRE-POST-treatment did not decrease SA-*β*-Gal levels more than PRE-treatment and POST-treatment conditions separately.

**Figure 8:**
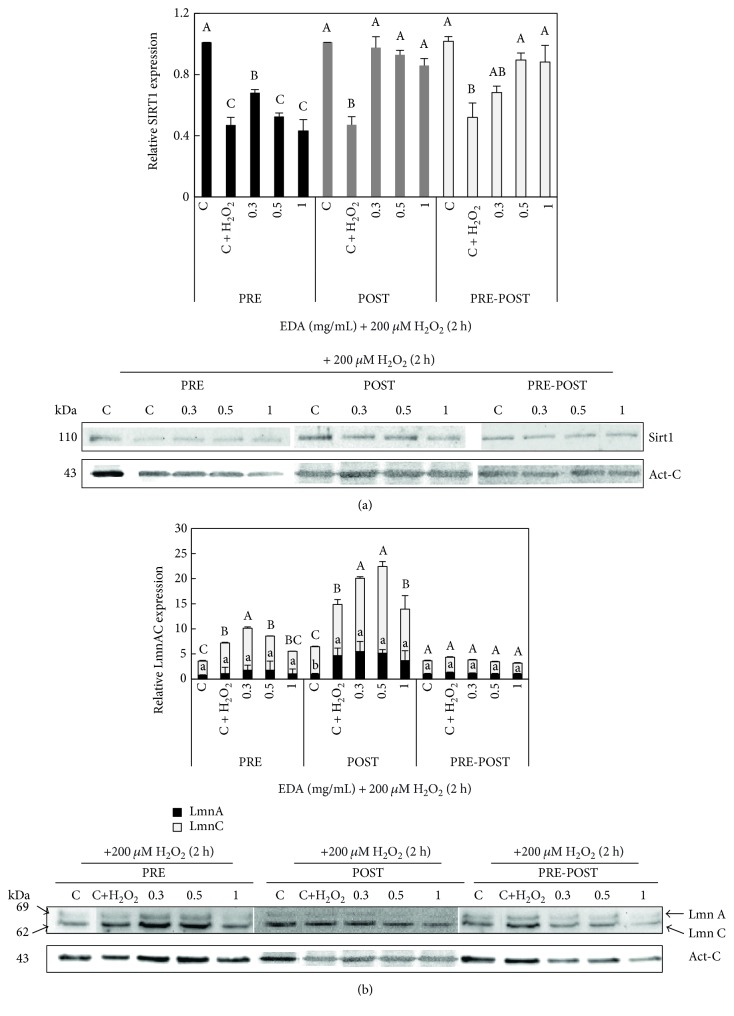
Effect of EDA in protein levels of Sirt1 and LmnA/C of SIPSF cells. (a) Densitogram (upper panel) and Western blot (lower panel) of Sirt1 and (b) LmnA/C in control (C, unexposed HFF cells), control 200 *μ*M H_2_O_2_-exposed cells (C + H_2_O_2_), and H_2_O_2_-exposed cells treated with 0.3, 0.5, and 1 mg/mL EDA. The time of EDA treatment was 24 h before H_2_O_2_ exposition (PRE), just after H_2_O_2_ exposition (POST), and after and before H_2_O_2_ exposition (PRE-POST). Capital letters indicate statistical comparative analysis among LmnA data and lowercase letters among LmnC. Each bar represents the mean ± S.E. of three replicates from three independent experiments, and samples that do not have a common letter are significantly different in each condition (PRE, POST, and PRE-POST) by Duncan's test at *P* < 0.05.

Regarding Sirt1, we observed significantly decreased levels in H_2_O_2_-exposed cells, compared to those in the control group. However, EDA treatments increased Sirt1 expression (Figure 8(a)). Specifically, the application of 0.3 mg/mL EDA prior H_2_O_2_ exposition increased Sirt1 expression whereas 0.5 and 1 mg/mL increased Sirt1 similarly in POST and PRE-POST conditions.

In case of LmnA/C, as it can be observed in Figure 8(b), both the PRE-treatment and POST-treatment of 0.3 mg/mL EDA induced an increase in LmnC levels, but not LmnA, which remained stable in every treatment comparing with SIPSF cells (C + H_2_O_2_). Strikingly, PRE-POST-treatment with EDA had no effects in protein LmnA/C contents.

These results indicate that different EDA doses have different effects on the onset of senescence markers in SIPSF cells. The morphology, SA-*β*-Gal, and contents of Sirt1 and LmnA/C suggested that treatment with 0.3 mg/mL EDA had a protective effect against H_2_O_2_ stress.

### 3.8. EDA Decreases the Expression of Trx2 in H_2_O_2_-Exposed Cells

**Figure 9:**
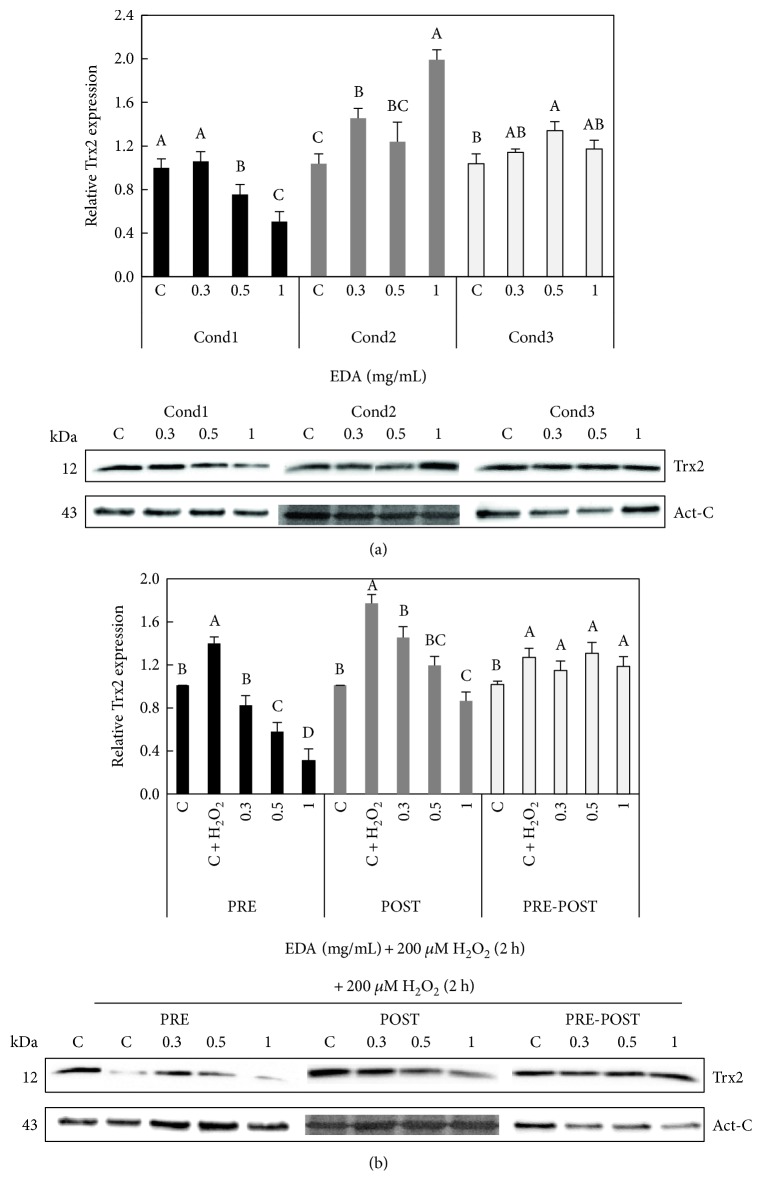
Effect of EDA over contents of Trx2 of HFF and SIPSF cells. (a) Densitogram (upper panel) and Western blot (lower panel) of Trx2 in HFF cells treated with different EDA doses. The time of EDA treatment was 24 h after seeding during 24 h (Cond1), 48 h after seeding during 24 h (Cond2), and 24 h after seeding during 48 h (Cond3). (b) Densitogram (upper panel) and Western blot (lower panel) of Trx2 in control (C, unexposed HFF cells), control 200 *μ*M H_2_O_2_-exposed cells (C + H_2_O_2_), and H_2_O_2_-exposed cells treated with 0.3, 0.5, and 1 mg/mL EDA. The time of EDA treatment was 24 h before H_2_O_2_ exposition (PRE), just after H_2_O_2_ exposition (POST), and after and before H_2_O_2_ exposition (PRE-POST). Each bar represents the mean ± S.E. of three replicates from three independent experiments, and samples that do not have a common letter are significantly different in each condition (Cond (a) or PRE, POST, and PRE-POST (b)) by Duncan's test at *P* < 0.05.

Finally, we investigated the effect of EDA treatment over unexposed *versus* H_2_O_2_-exposed cells in the conditions previously described (see Supplemental Figure S2). We checked that 0.5 and 1 mg/mL EDA decreased Trx2 contents in HFF cells in condition 1 but increased in condition 2 (Figure 9(a)). Levels of Trx2 were higher than those of the control in EDA-treated cells with 0.3 mg/mL in condition 2. In condition 3, only EDA treatment of 0.5 mg/mL increased levels of Trx2.

Besides, we observed that exposition to 200 *μ*M H_2_O_2_ increased Trx2 expression in HFF cells. PRE- and POST-treatment with 0.3, 0.5, and 1 mg/ml EDA significantly decreased (*P* < 0.05) Trx2 protein content compared to that of H_2_O_2_-exposed cells, whereas PRE-POST-treatment had no effects (Figure 9(b)). These experiments suggest that the protective effect of EDA may be unrelated to the role of Trx2 in the curbing ROS generation and/or their deleterious effects.

## 4. Discussion

Skin aging is a complex process influenced by intrinsic and extrinsic factors. Intrinsic factors such as genetic and metabolic aspects confer inevitable physiological changes over time. In contrast, extrinsic agents such as UV light exposure, extreme temperatures, pollution, or diet, among others, can accelerate the intrinsic senescence process [4, 28, 29]. During senescence, an increase in production of cellular reactive oxygen species (ROS) results in deleterious damages in different cell components. Antioxidants provide defense against ROS, and depletion in the levels of these compounds has been found in senescence cells *in vitro* as well as *in vivo* [30, 31]. In this context, the search for products and mainly natural products that can reduce oxidative stress and, hence, senescence induced by stressors is being object of increased interest in recent years.

Many natural products are endowed with regenerative properties as well as antioxidant activity. In the past, some of them have been identified in several products, such as, a secretion of the mollusk *Cryptomphalus aspersa* (SCA) [32], extracts from fern leaves [33], curcumin [34], green tea [35], or resveratrol [36]. In this study, we have tested the antisenescence effects of an extract from the Antarctic plant *Deschampsia antarctica* (EDA), an extremophile plant endowed with a high antioxidant capacity and with tested resistance to oxidant stressors such as UV radiation [37]. To carry out this work, we selected human foreskin fibroblasts as a model due to their importance in skin regeneration [4, 26], and as a trigger of stress-induced senescence, we used H_2_O_2_, which is known to be a ROS generator of premature aging [38, 39].

Premature senescence shares some characteristics with replicative or chronologic senescence, such as typical cell morphology, decreased cell proliferation up to irreversible growth arrest or increased SA-*β*-Gal levels. Morphological changes involved in senescence are a consequence of the cytoskeleton reorganization [4, 26]. *In vitro*, SHFF are much larger, displaying round and flat appearance compared to HFF. Moreover, H_2_O_2_-exposed cells were also similar to SHFF, whereas EDA PRE-treatment prevented the morphological changes associated to senescence. Indeed, EDA PRE-treatment of H_2_O_2_-exposed HFF cells maintained similar morphological characteristics (spindle shape and smaller size) to that of untreated HFF cells. This effect is consistent with that of other proven natural-origin antioxidants [4].

As previously indicated, senescence involves a decrease in cell proliferation until growth arrest. In this regard, PCNA is a marker of cell proliferation that decreases its expression with age [40]. Some antioxidants such as genistein increase PCNA contents, preserving cutaneous proliferation [7]. Likewise, other antioxidant agents, for example, SCA, resveratrol, caffeic acid, or ascorbic acid, have been described to increase cell proliferation, and this effect seems to be related to the repair ability of each compound [26, 32, 41, 42]. Here, we demonstrate that EDA promotes a slight increase in HFF proliferation and viability, which indicates the proliferative effect of this compound.

The effects of H_2_O_2_ over cell proliferation as inductor of oxidative damage have been broadly studied in several cell types [28, 43–45]. We report that EDA PRE-treatment prevents the antiproliferative effect of H_2_O_2_, moreover, increasing cell proliferation over control non-H_2_O_2_-exposed cells. This behavior was similar to that found in other antioxidant compounds [20, 33, 46]. In fact, EDA extracts are rich in phenolic substances including flavonoids such as apigenin and luteolin, suggesting that the protective role of EDA may be related to its antioxidant capability. Nevertheless, this role would need further studies. Additional mechanisms may include an effect on the cellular levels of DNA-modifying enzymes, for example, Sirt1. Sirt1 is a nicotinamide adenine dinucleotide- (NAD^+^-) dependent deacetylase and ADP-ribosyltransferase that is involved in mitochondrial biogenesis and DNA modification. Importantly, transgenic mice for Sirt1 display a significantly longer life span and delayed onset of senescence, including improved physical parameters [47]. At a molecular level, Sirt1 promotes cell proliferation and its expression levels decreased with cellular senescence [48, 49]. Furthermore, deacetylation of FOXO, p53, p73, Ku70, and Smad7 proteins by Sirt1 induced tolerance to oxidative stress and inhibited apoptosis [50, 51]. Different doses of EDA negated the inhibition of Sirt1 expression caused by H_2_O_2_, which partly explains the positive effect of EDA protecting against senescence via increased resistance to oxidative stress [52]. In this regard, EDA was also effective in preventing other markers of stress-induced senescence, for example, increased SA-*β*-Gal, which is a commonly used marker of cell senescence [53, 54].

It has been showed that a significant negative correlation exists between the percentage of senescent cells and the percentage of proliferating cells during human disc tissue degeneration [55]. However, this relationship is not always so; in some cases, an excessive mitogenic stimulation may induce senescence [56]. These reports underline that EDA decreases senescence markers, but this fact is not completely parallel to an increase in cell proliferation.

We find that the increase of Trx2 after the exposition of HFF to H_2_O_2_ is counteracted by the EDA treatment, indicating that EDA could promote protection against H_2_O_2_ premature-induced senescence decreasing the levels of oxidative stress. Recent studies have shown that Trx2 overexpression *in vitro* can protect mammalian cells against t-butyl hydroperoxide and etoposide-induced apoptosis, as well as increasing the mitochondrial membrane potential [57, 58]. In addition, human cardiomyocytes deficient in Trx2 display increased cellular ROS and apoptosis [23]. On the other hand, overexpression of either *Trx1* or *Trx2* has been shown to influence life span in experimental models [24, 59]. The increase in Trx1, Trx2, and thioredoxin reductase proteins has been proposed as a compensatory response to counteract increased oxidative stress during the senescence process [60].

EDA also prevents the stress-induced decrease in proteins that control the nuclear architecture, for example, lamins A and C. These proteins are key players in cellular senescence, as illustrated by their genetic depletion, which causes early onset of senescence, comprising a group of rare genetic diseases collectively referred to as laminopathies. In natural senescence, LmnA seems to remain constant over time whereas LmnC decreases significantly [17, 18], although this could be cell-type dependent, since other studies report a concomitant decrease of both isoforms [17]. In our experiments, H_2_O_2_ decreased LmnC and LmnA, and this decrease was counteracted by the PRE-treatment with EDA, which is consistent with an antisenescence effect of EDA in H_2_O_2_-exposed HFF cells.

In conclusion, our data proves the positive effect of an extract from *Deschampsia antarctica* to counteract H_2_O_2_ stress-induced premature senescence in human diploid foreskin fibroblasts suggesting its potential as a therapeutic agent for treating skin aging.

## Supplementary Material

FIGURE S1: EDA treatments along the cell cycle. (1) HFF cells were seeding in 96 well plates at a density of 3.5 × 10^3^ cells/cm^2^. (2) 24 h after seeding different EDA doses were applied during 24 h (cond1) and 48 h (cond3). (3) 48 h after seeding, HFF cells (50% confluency) were treated with EDA during 24 h (cond2), whereas in cells from cond1, EDA was removed and fresh culture medium was applied. (4) 72 h after seeding different parameters were evaluated. FIGURE S2: EDA treatments along the cell cycle in H2O2-exposed cells. (1) HFF cells were seeding in 96 well plates at a density of 3.5 × 10^3^ cells/cm^2^ (2) 24 h after seeding different EDA doses were applied during 24 h (PRE) and 48 h (PRE-POST). (3) 48 h after seeding cell cultures were washed with PBS and incubated during 30min-2h with different doses of H_2_O_2_. (4) After H_2_O_2_ incubation new EDA product was added to PRE-POST and POST cultures. (5) 24 and 48 h after H_2_O_2_ exposition different parameters were evaluated.

## Abbreviations

SA-*β*-Gal: Senescence-associated beta-galactosidase
DMEM: Dulbecco's modified Eagle's medium
EDA: Extract from Deschampsia antárctica
EDTA: Ethylenediaminetetraacetic acid
FDG: Fluorescein di-*β*-galactopyranoside
HFF: Human foreskin fibroblasts
LmnA/C: Lamin A/C
MTT: 3-(4,5-Dimethylthiazol-2-yl)-2,5-diphenyltetrazolium bromide
PCNA: Proliferating cell nuclear antigen
PD: Population doubling
SHFF: Senescence human foreskin fibroblasts
SIPS: Stress-induced premature senescence
SIPSF: Stress-induced premature senescence fibroblasts
Sirt: Sirtuin.
