# Supplementary material for: An Extract from the Plant Deschampsia antarctica Protects Fibroblasts from Senescence Induced by Hydrogen Peroxide
Source: Oxid Med Cell Longev. 2017 Aug 15;2017:2694945. doi: 10.1155/2017/2694945 (PMC5574316; doi:10.1155/2017/2694945)
Supplement: Supplementary file 1 — FIGURE S1: EDA treatments along the cell cycle. (1) HFF cells were seeding in 96 well plates at a density of 3.5 × 10^3^ cells/cm2. (2) 24 h after seeding different EDA doses were applied during 24 h (cond1) and 48 h (cond3). (3) 48 h after seeding, HFF cells (50% confluency) were treated with EDA during 24 h (cond2), whereas in cells from cond1, EDA was removed and fresh culture medium was applied. (4) 72 h after seeding different parameters were evaluated. FIGURE S2: EDA treatments along the cell cycle in H2O2-exposed cells. (1) HFF cells were seeding in 96 well plates at a density of 3.5 × 10^3^ cells/cm2 (2) 24 h after seeding different EDA doses were applied during 24 h (PRE) and 48 h (PRE-POST). (3) 48 h after seeding cell cultures were washed with PBS and incubated during 30min-2h with different doses of H2O2. (4) After H2O2 incubation new EDA product was added to PRE-POST and POST cultures. (5) 24 and 48 h after H2O2 exposition different parameters were evaluated. [file 2694945.f1.pdf]

FIGURE S1

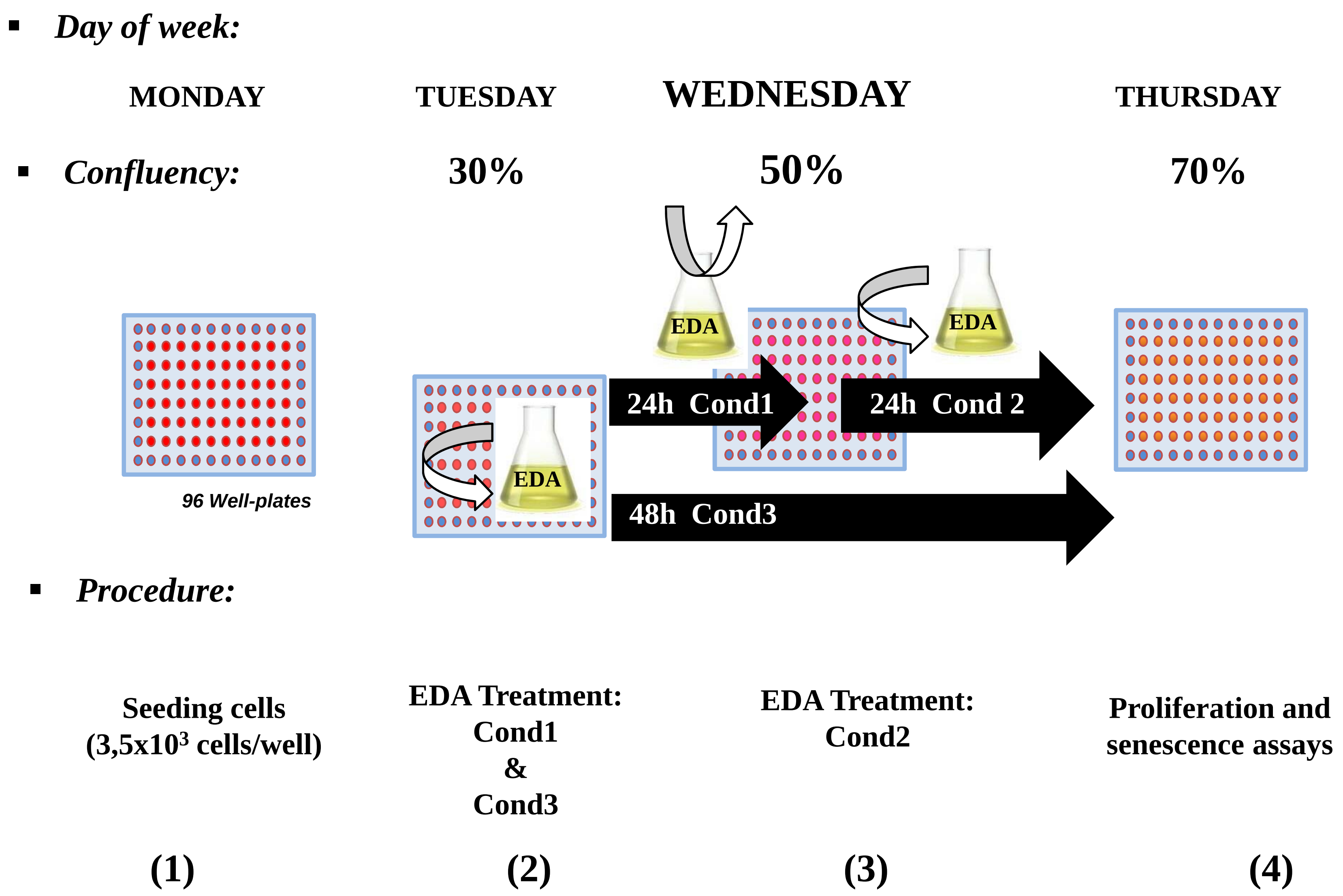

Day of week:
MONDAY
TUESDAY
WEDNESDAY
THURSDAY
Confluency:
30%
50%
70%
EDA
EDA
EDA
96 Well-plates
24h Cond1
24h Cond 2
48h Cond3
Procedure:
Seeding cells (3,5x10³ cells/well)
EDA Treatment: Cond1 & Cond3
EDA Treatment: Cond2
Proliferation and senescence assays
(1)
(2)
(3)
(4)

FIGURE S2

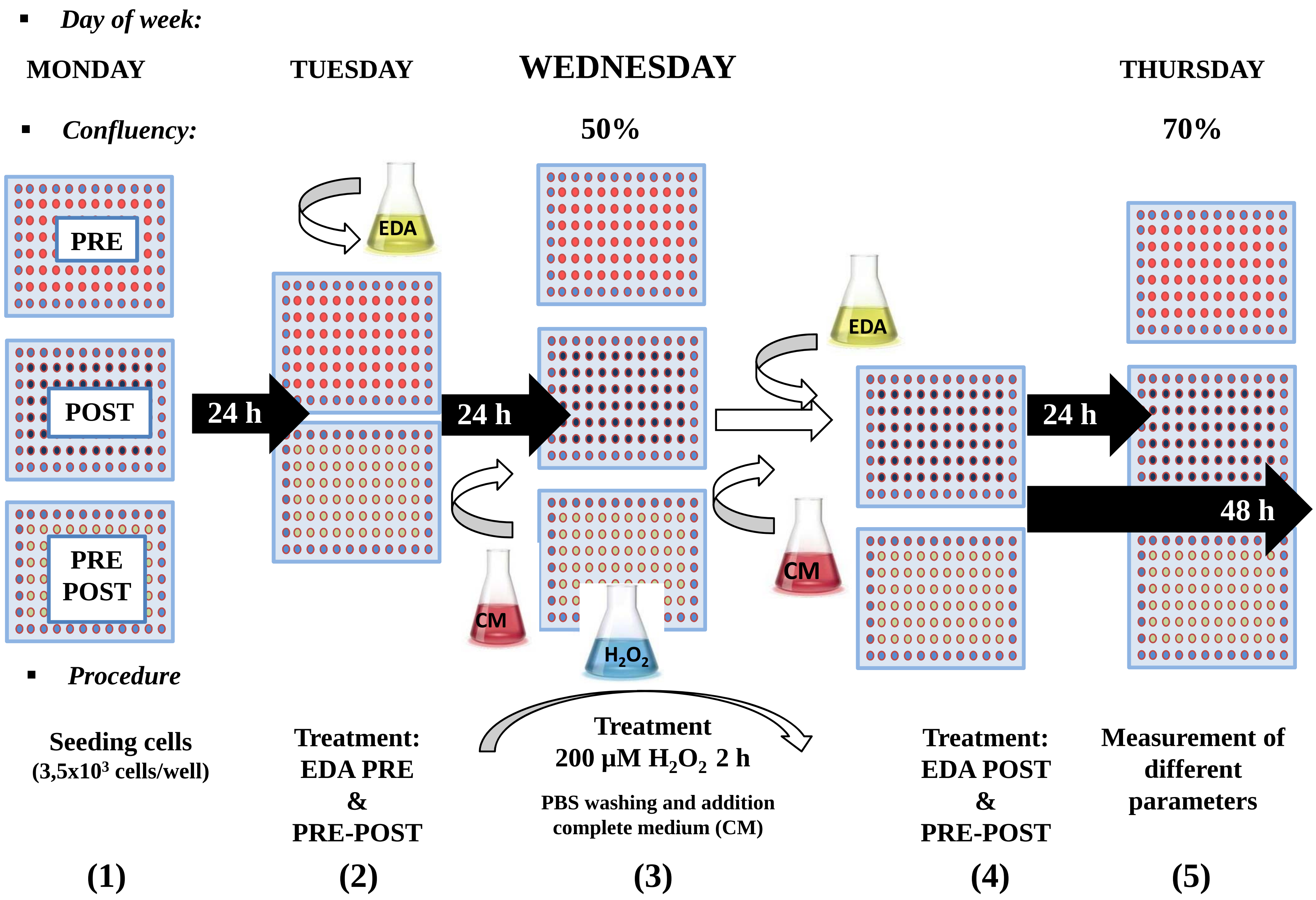

Day of week:
MONDAY
TUESDAY
WEDNESDAY
THURSDAY
Confluency:
50%
70%
PRE
POST
PRE POST
24 h
EDA
24 h
CM
H2O2
EDA
CM
24 h
48 h
Procedure
Seeding cells (3,5x10^3 cells/well)
Treatment: EDA PRE & PRE-POST
Treatment 200 µM H2O2 2 h
PBS washing and addition complete medium (CM)
Treatment: EDA POST & PRE-POST
Measurement of different parameters
(1)
(2)
(3)
(4)
(5)
